# Optical Coherence Tomography for Invasive Oral Squamous Cell Carcinoma: Diagnostic Accuracy and Grade- and Subsite-Associated Imaging Features

**DOI:** 10.3390/jcm15031102

**Published:** 2026-01-30

**Authors:** Waseem Jerjes, Zaid Hamdoon, Dara Rashed, Colin Hopper

**Affiliations:** 1 Faculty of Medicine, Imperial College London, London W12 0BZ, UK; 2 College of Dental Medicine, University of Sharjah, Sharjah 27272, United Arab Emirates; 3 Unit of OMFS, UCL Eastman Dental Institute, London WC1E 6DE, UK; d.rashed@alumni.ucl.ac.uk (D.R.); c.hopper@ucl.ac.uk (C.H.)

**Keywords:** optical coherence tomography, oral squamous cell carcinoma, tumour grading, diagnostic imaging, surgical margins

## Abstract

**Background**: Early and accurate diagnosis remains crucial to improving outcomes in oral cancer. Optical coherence tomography (OCT) offers real-time, high-resolution imaging that may support diagnosis and treatment planning in oral squamous cell carcinoma (OSCC). **Methods**: In this prospective study, preoperative OCT scans were obtained from 68 histologically confirmed OSCC lesions, with 30 paired adjacent mucosa samples from the same patients as histologically negative comparators (diagnostic dataset: 98 lesions). OCT findings were compared with histopathology for diagnostic performance, OCT biomarker patterns by tumour grade, tumour depth measurement, margin assessment, and subsite-specific performance. **Results**: OCT demonstrated 98.5% sensitivity, 96.7% specificity, and an AUC of 0.98 for detection of invasive OSCC. OCT biomarkers—including abnormal epithelial architecture with variable epithelial thickness, stratification loss, basement membrane disruption, and increased subepithelial reflectivity—varied systematically with tumour differentiation grade. Tumour depth measurements showed acceptable agreement with histology, while margin definition was correct in 80% of cases. Performance was highest in the tongue and the floor of the mouth, with reduced performance in posterior/keratinised subsites. Image artefacts occurred in 5.1% of scans. **Conclusions**: OCT provides a reproducible, real-time adjunct for diagnosis, margin planning, and lesion stratification in OSCC, with recognised limitations related to light attenuation and operator-dependent factors. Multicentre validation and integration with digital interpretation platforms are warranted.

## 1. Introduction

Oral squamous cell carcinoma (OSCC) is the most common malignancy of the oral cavity, accounting for more than 90% of oral cancers worldwide. Globally, OSCC remains a major health burden, with 377,713 new cases and 177,757 deaths reported in 2020 (GLOBOCAN) [1]. Despite advances in surgery, radiotherapy, and systemic therapies, five-year survival has remained broadly static at approximately 50–60%, largely due to late presentation, regional metastasis, and the clinical challenge of achieving adequate surgical margins [2,3]. Earlier diagnosis and more accurate delineation of tumour extent are therefore central to improving outcomes.

Clinically, suspected OSCC is typically assessed through a combination of careful oral examination (including palpation and assessment of induration/fixity), risk factor evaluation, and prompt incisional biopsy for histopathological confirmation and grading. Cross-sectional imaging is used for local staging and nodal assessment (with MRI/CT and, where appropriate, ultrasound-guided nodal evaluation), and PET-CT may be used in selected cases for advanced disease [1,2,3]. Standard treatment is usually surgical resection with appropriate reconstruction and neck management, with adjuvant radiotherapy or chemoradiotherapy guided by pathological risk features (e.g., positive/close margins, extranodal extension, perineural or lymphovascular invasion). Prognosis varies by stage and nodal status, and delayed presentation remains a principal driver of poorer outcomes. Common differentials for suspicious oral lesions include traumatic/irritational ulceration, chronic inflammatory lesions, oral potentially malignant disorders (e.g., leukoplakia/erythroplakia with dysplasia), lichen planus/lichenoid reactions, candidiasis, and verrucous or other epithelial proliferations [4,5].

Histopathological assessment of a tissue biopsy remains the gold standard for diagnosis and grading of OSCC. However, biopsy-based diagnosis has recognised limitations, including sampling error, interobserver variability, and patient discomfort related to an invasive procedure [4,5]. In routine practice, biopsies often sample only part of a heterogeneous lesion, which can underestimate the true extent of disease or miss the most biologically aggressive component [6]. These limitations are particularly relevant when clinical decisions depend on margin planning, selection of biopsy sites, or rapid risk stratification in patients with complex or multifocal disease.

Optical coherence tomography (OCT) is a non-invasive imaging modality capable of generating cross-sectional images of tissue microstructure in real time. Using near-infrared light, OCT can achieve micrometre-scale resolution and visualise epithelial architecture, basement membrane (BM) integrity, and subepithelial stromal features—structures commonly altered in OSCC [7,8]. In oral oncology, OCT has potential applications beyond detection alone, including guidance of biopsy to the most diagnostically informative regions, adjunctive assessment of tumour margins, and support for intraoperative decision-making [9,10]. These use cases are clinically attractive because they address practical problems encountered in both diagnostic clinics and surgical pathways.

Prior studies have demonstrated that OCT can detect dysplastic change and help distinguish benign from malignant mucosal lesions. Reported optical biomarkers include epithelial thickening, increased scattering, loss of stratification, and disruption or loss of the BM, with several studies showing correlation between OCT appearance and histopathological features of malignancy [11,12]. However, the published evidence base remains heterogeneous in study design, reference standards, and case mix, and many reports include relatively small sample sizes or mixed diagnostic categories. Importantly, robust evaluation of OCT specifically in confirmed OSCC cohorts—together with systematic analysis of performance by anatomical subsite and characterisation of imaging features across tumour differentiation grades—has remained limited [13,14,15,16,17]. The subsite is clinically important because keratinised or posterior regions may attenuate the OCT signal and reduce interpretability, potentially affecting diagnostic accuracy and reliability in real-world settings.

This prospective single-centre clinical study therefore evaluated OCT in 68 histologically confirmed OSCC lesions, with 30 paired adjacent mucosa samples acquired from the same patients to provide histologically negative comparators for diagnostic performance analysis (diagnostic dataset: 98 lesions). The primary objectives were (1) to determine the diagnostic performance of OCT for invasive OSCC compared with histopathology; (2) to evaluate OCT-based margin delineation against surgical histology; (3) to examine OCT imaging biomarkers associated with tumour differentiation grade; and (4) to explore subsite-specific performance across the oral cavity. By integrating lesion profiling with quantitative OCT feature assessment, this study aims to clarify the clinical utility of OCT as an adjunct tool in oral oncology, with a view to improving diagnostic confidence, supporting margin planning, and informing future integration with digital interpretation platforms and multicentre validation.

## 2. Materials and Methods

### 2.1. Study Design and Ethical Approval

This prospective, single-centre study was conducted at the Head and Neck Oncology Unit, UCL Eastman Dental Institute, London. Participant recruitment and OCT imaging were conducted between [March 2018] and [January 2022]. The objective was to evaluate the diagnostic accuracy of OCT in the assessment of OSCC, with a particular focus on lesion characterisation, margin delineation, quantitative depth measurement, and anatomical subsite performance.

The study was performed in accordance with the Declaration of Helsinki and approved by the Moorfields & Whittington Local Research Ethics Committee (REC reference: 07/Q0504/4). All participants received written and verbal information and provided written informed consent. Data were anonymised at source and stored on password-protected systems.

### 2.2. Study Population

Sixty-eight consecutive adult patients (≥18 years) were recruited from the outpatient oral oncology clinic. Eligible participants had a clinically apparent lesion suspicious for malignancy, had not received prior oncological therapy, and were suitable for OCT imaging and definitive surgical excision. Exclusion criteria included recurrent tumours, prior head and neck radiotherapy, and patients deemed medically unfit for surgery.

Demographic and clinical variables were recorded, including age, sex, risk factors (smoking, alcohol, betel nuts), comorbidities, and lesion characteristics (site, size, and clinical morphology).

To permit estimation of specificity within the same clinical cohort, histologically negative adjacent mucosa samples were included as comparators. Adjacent mucosa was acquired from clinically normal-appearing tissue adjacent to the primary lesion, scanned within the same visit and confirmed histologically negative. Each patient contributed one OSCC lesion (disease-positive); where adjacent mucosa was available, one comparator sample per patient was included. The diagnostic performance dataset therefore comprised 98 lesions/samples (i.e., one representative scan per lesion/sample for analysis): 68 OSCC (disease-positive) and 30 adjacent mucosa samples (disease-negative).

### 2.3. OCT Imaging Protocol

In vivo imaging was performed using a multi-beam swept-source OCT system (VivoSight^®^, Michelson Diagnostics Ltd., Kent, UK; axial resolution 5 µm; lateral resolution 7.5 µm), optimised for mucosal imaging. OCT scans were obtained pre-biopsy or pre-resection. Imaging was performed by two operators who were blinded to histopathological diagnosis at the time of acquisition. Each target region was scanned multiple times to ensure adequate coverage and reproducibility; for quantitative scoring and diagnostic accuracy analyses, one representative scan per target region (least artefact and best visualisation of the lesion–normal transition) was selected for evaluation. To minimise potential selection bias, scan selection followed this predefined quality criterion, image artefact/clarity was recorded prospectively, and interpretation was performed blinded and in duplicate.

For OSCC lesions, the clinically visible lesion and a 2–3 mm peripheral zone of apparently normal mucosa were scanned. For the diagnostic comparator group, adjacent mucosa was scanned from clinically normal-appearing tissue near the lesion. OCT images were assessed for predefined features including epithelial thickness, epithelial stratification/architecture, BM visibility and disruption, subepithelial stromal changes, and signal attenuation/artefacts. OCT feature assessment was performed by two experienced clinicians blinded to histopathological outcomes. Disagreement was resolved by consensus discussion.

Epithelial thickness was measured on OCT using the system callipers on the representative scan, from the mucosal surface to the most discernible epithelial–stromal interface (BM-like boundary where visible). To account for heterogeneity, three measurements were taken across the region of interest (centre and two adjacent points spanning the lesion where an intact surface epithelium was present) and averaged. In ulcerated or markedly disorganised areas, measurements were taken from the nearest intact epithelium adjacent to the ulcerated zone and interpreted as an architectural marker rather than uniform ‘thickening’.

For margin assessment, OCT was used to delineate the superficial (lateral) lesion extent by identifying a transition zone from the abnormal epithelium to apparently normal mucosa within the scanned 2–3 mm peripheral zone. The margin assessment was confined to the mucosal surface and immediate subepithelial compartment within OCT penetration limits (~1.5–2 mm); deep margin assessment was not undertaken. Accordingly, OCT was not used to assess deep margins beyond approximately 2 mm from the mucosal surface. No fixed distance-based threshold (e.g., mm from the visible lesion edge) was prespecified; assessment was based on the OCT-defined transition zone within the scanned peripheral field.

For avoidance of doubt, all OSCC cases in this study were histologically invasive (i.e., BM breach present by definition); “BM visibility” and “BM disruption” refer to OCT interpretability of the BM-like interface (discernible linear band and its continuity), which may be reduced by keratinisation/attenuation and does not imply an intact BM in invasive lesions.

An ordinal/continuous OCT malignancy score was constructed a priori by combining graded OCT features recorded on the representative scan for each lesion/sample. The composite score used equal-weighted components reflecting epithelial and subepithelial disruption: loss of epithelial stratification (0 = absent, 1 = present), reduced basement membrane (BM) visibility (0 = visible, 1 = not visible), BM disruption (0 = absent, 1 = present), subepithelial reflectivity (0–5), and image clarity/attenuation (0–5). For the composite score, the image clarity rating was directionally aligned so that higher values reflected greater abnormality (attenuation component = 5 − clarity rating). Component values were summed to yield a continuous score (range 0–13), which was used to generate ROC curves and estimate AUC.

### 2.4. Surgical Resection and Histopathological Analysis

Following OCT scanning, all patients underwent definitive lesion excision under local or general anaesthesia. Resected specimens were orientated, inked, and fixed in 10% neutral buffered formalin (routine pathology supply, UCLH/UCL Eastman Dental Institute, London, UK). Two oral and maxillofacial pathologists, blinded to OCT findings, performed standardised histopathological assessment.

No specimen-specific contraction factor was measured; therefore, histopathological measurements were reported as per routine pathology without numerical correction for formalin-associated shrinkage [18].

Tumour differentiation was graded using World Health Organization criteria as well, moderately, or poorly differentiated OSCC. Tumour depth (measured from the epithelial surface to the deepest point of invasion), perineural invasion, lymphovascular invasion, and margin status were recorded. Histopathology served as the reference standard for all diagnostic accuracy analyses and for comparison of OCT-derived tumour depth and margin assessments.

### 2.5. Outcomes and Statistical Analysis

The primary outcome was diagnostic performance of OCT for identifying invasive OSCC using histopathology as the reference standard. All diagnostic performance analyses were conducted at the lesion/sample level (one representative scan per lesion/sample). Diagnostic accuracy was summarised using sensitivity, specificity, positive predictive value (PPV), negative predictive value (NPV), accuracy, and AUC, derived from the lesion-level contingency table and ROC analysis. Ninety-five per cent confidence intervals (95% CIs) were calculated for sensitivity and specificity using exact binomial methods and for AUC using a standard error-based approach (Hanley–McNeil). Subsite performance was evaluated descriptively using lesion-level anatomical distributions. Image artefacts were recorded and expressed as a proportion of the analysed OCT scans.

For exploratory subsite-specific ROC analyses, AUCs were accompanied by approximate 95% CIs using the same standard error-based approach, recognising the small numbers in some subsites and the shared comparator set. Formal between-operator variability in acquisition was not prespecified for hypothesis testing; to mitigate potential operator effects, acquisition followed a standardised protocol and image artefact/clarity were recorded, with interpretation performed blinded and in duplicate.

Agreement between OCT and histology for tumour depth measurement was assessed using Bland–Altman analysis. Agreement between OCT lesion-level classification (invasive vs non-invasive) and histopathology was evaluated using Cohen’s kappa (κ). Subgroup analyses were performed according to lesion subsite and histopathological grade. Statistical analyses were conducted using SPSS v28.0 (IBM Corp., Armonk, NY, USA), with *p* < 0.05 considered statistically significant.

## 3. Results

The study included 68 patients (mean age 59.2 ± 10.3 years; range 34–83), with 40 males and 28 females. Key risk factors included smoking status (current/former/never: 30/20/18), alcohol use (42/26 yes/no), and betel nut use (10/58 yes/no), with common comorbidities including hypertension (29.4%), diabetes (22.1%), and cardiovascular disease (17.6%) (Table 1). Across 68 OSCC lesions, the mean lesion size was 12.4 ± 4.7 mm (range 5–28 mm), and lesions were most commonly ulcerated (50.0%) or exophytic (29.4%) (Table 2). The most frequent anatomical subsites were the tongue (38.2%), the floor of the mouth (20.6%), and the buccal mucosa (17.6%), with histopathology showing predominantly well- (41.2%) and moderately differentiated (44.1%) OSCC and 14.7% poorly differentiated tumours; perineural invasion was present in 27.9% and lymphovascular invasion in 17.6% (Table 2).

### 3.1. Diagnostic Accuracy of OCT in Detecting Invasive OSCCs

**Figure 1:**
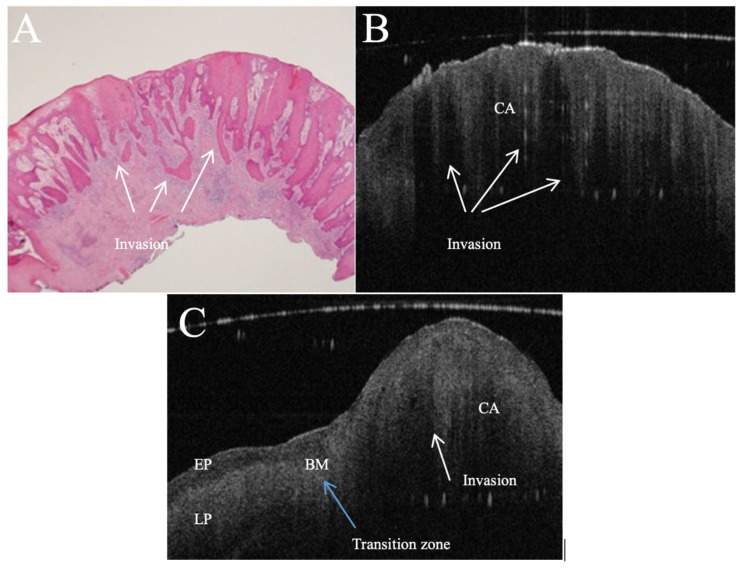
In vivo OCT and histopathology images (**A**,**B**) (H&E, original magnification ×10 and ×20) of an erythro-leukoplakic lesion on soft palate revealing multifocal carcinoma (CA). OCT image matches histopathology in displaying multifocal epithelial down growth and invasion into the subepithelial layers (white arrows). Furthermore, the basement membrane is indiscernible through the entire OCT scan as a coherent prominent landmark. The bottom image (**C**) is an in vivo OCT image of a transition zone (blue arrow) between healthy tissue and invasive carcinoma (CA) invading through the basement membrane. It also shows normal-thickness stratified squamous epithelium, which is darker compared to the homogeneous lamina propria; while crossing the transition zone, epithelial downgrowth and invasion can be clearly recognised, and the lamina propria become non-homogeneous. BM = basement membrane; EP = epithelium; LP = lamina propria; CA = invasive carcinoma.

All 68 patients underwent successful preoperative OCT imaging (Figure 1). For diagnostic accuracy analysis, 30 histologically negative adjacent mucosa samples acquired from a subset of the same patients were included as comparators, giving a diagnostic dataset of 98 lesions/samples (one representative scan per lesion/sample): 68 OSCCs and 30 adjacent mucosa samples (Table 3).

**Figure 2:**
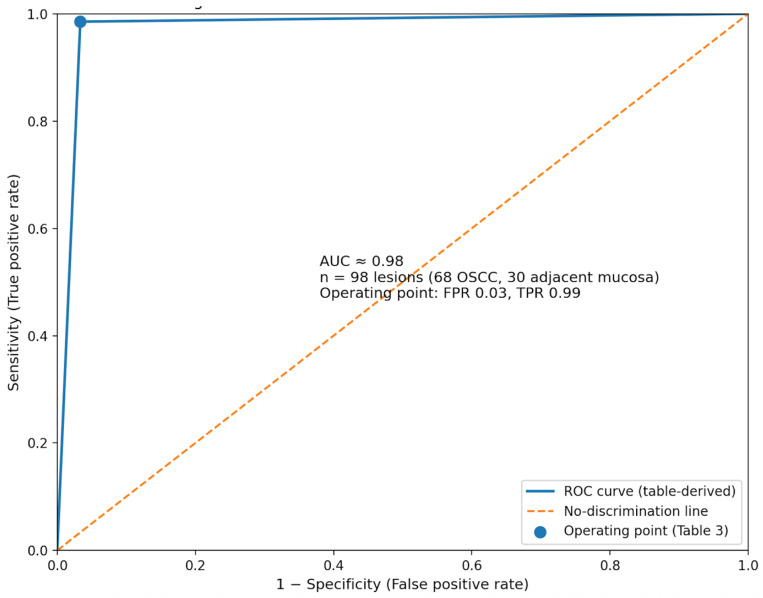
ROC curve for OCT detection of invasive OSCC. Receiver operating characteristic (ROC) analysis demonstrates strong discrimination of invasive OSCC by OCT (AUC ≈ 0.98) in a diagnostic dataset of 98 lesions (68 OSCC; 30 adjacent mucosa). The solid blue line represents the ROC curve, and the dashed diagonal indicates no-discrimination performance. The marker denotes the operating point corresponding to the contingency table performance, with the false positive rate (FPR) 0.03 and true positive rate (TPR) 0.99.

Using histopathology as the reference standard, OCT correctly identified invasive OSCC in 67/68 cases (true positives), with 1 false negative, and correctly classified 29/30 adjacent mucosa samples as negative (true negatives), with 1 false positive. This corresponded to 98.5% sensitivity (95% CI: 92.1–100.0), 96.7% specificity (95% CI: 82.8–99.9), 98.5% PPV, 96.7% NPV, and 98.0% overall accuracy (Table 3). ROC analysis based on the ordinal/continuous OCT score demonstrated strong classification performance with an AUC of 0.98 (95% CI: 0.96–1.00) (Figure 2). Image artefact affecting interpretation was recorded in 5/98 scans (5.1%). Agreement between OCT lesion-level classification and histopathology was excellent (Cohen’s κ = 0.952; 95% CI: 0.839–1.000).

**Figure 3:**
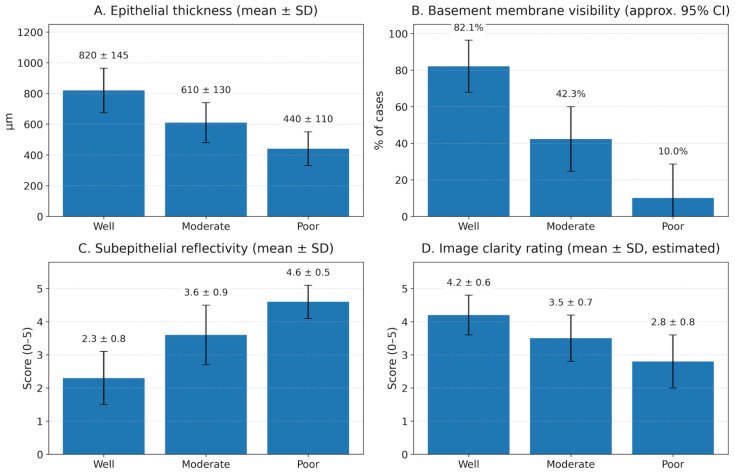
OCT imaging features by OSCC grade. Four-panel bar chart showing OCT-derived biomarkers across histopathological differentiation grades (well, moderately, and poorly differentiated OSCC; n = 28, 30, and 10, respectively). (**A**) Mean epithelial thickness (μm) ± SD. (**B**) Basement membrane visibility (%) with approximate 95% confidence intervals for proportions. (**C**) Mean subepithelial reflectivity score (0–5) ± SD. (**D**) Mean image clarity rating (0–5) shown as mean ± SD.

Across OSCC lesions, OCT frequently demonstrated imaging features consistent with invasion and microstructural disruption, including an abnormal epithelial architecture with variable epithelial thickness, loss of stratification, basement membrane disruption, and increased subepithelial reflectivity (Figure 3; Table 4). The single false negative case occurred in a lesion located on the hard palate/gingiva region, where keratinisation and attenuation reduced visualisation of subtle basement membrane disruption. The single false positive classification was attributable to pseudo-loss of basement membrane under hyperkeratosis, consistent with the most common false positive feature identified.

Interobserver agreement for OCT interpretation was high, indicating substantial agreement between readers for lesion-level classification (invasive vs. non-invasive). Disagreements were infrequent and were resolved by consensus before final reporting.

### 3.2. OCT Margin Assessment Versus Histopathology

All 68 OSCC lesions underwent OCT evaluation for lesion-to-normal delineation prior to definitive excision. Margin delineation was assessed at the lateral (mucosal) interface only by sweeping the probe across the clinically visible lesion edge into adjacent clinically normal mucosa. A margin assessment was defined as ‘correct’ when the OCT-defined lesion–normal transition corresponded with histopathology at the mucosal margin (tumour present on the lesion side and absent in the adjacent mucosa at the corresponding margin). Using this operational definition, OCT margin delineation agreed with histopathological margin assessment in 80% of cases. Mismatches were most commonly associated with reduced penetration and interpretability in thicker or more keratinised subsites and in areas with inflammation-related scattering.

### 3.3. Imaging Biomarkers and LESION Stratification by Tumour Grade

OCT-derived imaging biomarkers varied systematically with histopathological differentiation grade (Table 4; Figure 3). The mean epithelial thickness decreased from 820 ± 145 µm (well-differentiated; n = 28) to 610 ± 130 µm (moderately differentiated; n = 30) and 440 ± 110 µm (poorly differentiated; n = 10). Loss of epithelial stratification increased with worsening differentiation (21.4%, 63.3%, 100%). Basement membrane visibility decreased (82.1%, 42.3%, 10.0%), while basement membrane disruption increased (17.9%, 57.7%, 90.0%). Subepithelial reflectivity increased (2.3 ± 0.8, 3.6 ± 0.9, 4.6 ± 0.5) with higher signal attenuation and reduced tumour–stromal interface clarity (clear in 85.7%, 60.0%, 30.0%, respectively), alongside lower overall image clarity ratings (4.2, 3.5, 2.8). Epithelial thickness on OCT was interpreted as a marker of architectural distortion rather than uniform thickening and may appear reduced in higher-grade tumours due to loss of organised stratified layers and surface ulceration.

These grade-associated differences were statistically significant. Epithelial thickness differed across grades (one-way ANOVA, *p* < 0.001) and subepithelial reflectivity also differed across grades (one-way ANOVA, *p* < 0.001). Categorical OCT features varied by grade (χ^2^ tests): stratification loss (*p* < 0.001), basement membrane visibility (*p* < 0.001), basement membrane disruption (*p* < 0.001), and tumour–stromal interface clarity (*p* = 0.004).

Because all included tumours were histologically invasive, these BM metrics reflect OCT visualisation of the epithelial–stromal interface (discernibility and continuity) rather than the presence or absence of true BM breach.

### 3.4. Quantitative Agreement for Tumour Depth Estimation

**Figure 4:**
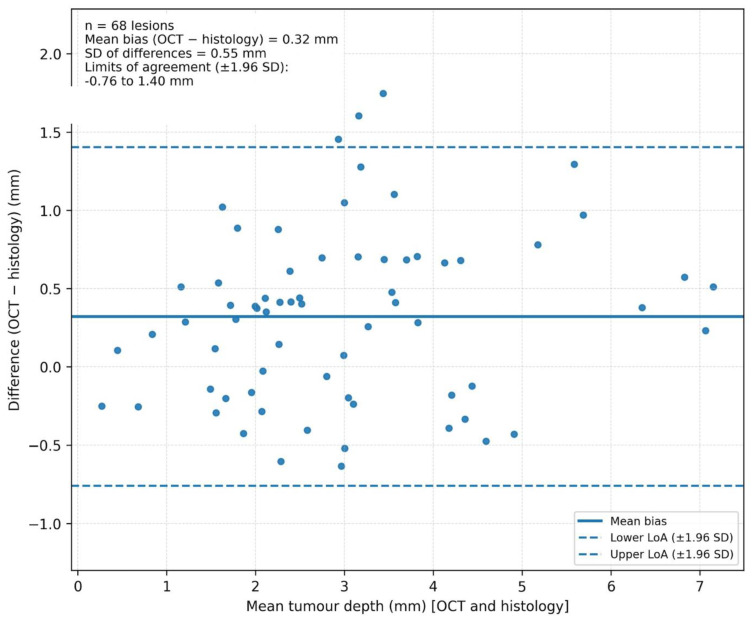
Bland–Altman plot comparing tumour depth measurements by OCT versus histology. Scatter plot of the difference in tumour depth measurements (OCT−histology) against the mean tumour depth for 68 OSCC lesions. The solid horizontal line indicates the mean bias (OCT overestimation), and the dashed lines represent the 95% limits of agreement (mean bias ± 1.96 SD). This analysis demonstrates acceptable agreement between OCT-derived and histology-derived depth measurements, supporting OCT’s quantitative reliability for depth estimation within its penetration limits.

Bland–Altman analysis of tumour depth measurements (n = 68 OSCC lesions) showed a small positive mean bias (OCT−histology) of 0.32 mm (SD of differences 0.55 mm), indicating slight OCT overestimation. The 95% limits of agreement were −0.76 to 1.40 mm (Figure 4).

### 3.5. Anatomical Subsite Variation in OCT Performance and Feature Detection

**Figure 5:**
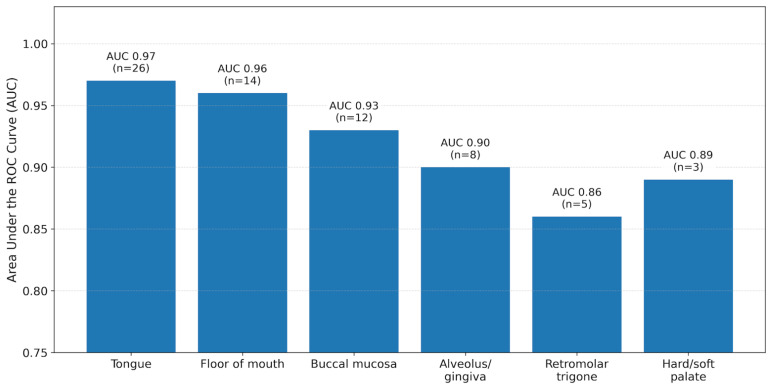
OCT diagnostic performance (AUC) by anatomical subsite. Bar chart showing the area under the ROC curve (AUC) for OCT detection of invasive OSCC across oral subsites, with approximate 95% confidence intervals for AUC. The highest performance is observed in the tongue and the floor of the mouth, with comparatively reduced performance in posterior and/or keratinised regions such as the retromolar trigone and palate/gingiva. Subsite-specific AUC confidence intervals should be interpreted cautiously given the small numbers and the shared comparator set.

**Figure 6:**
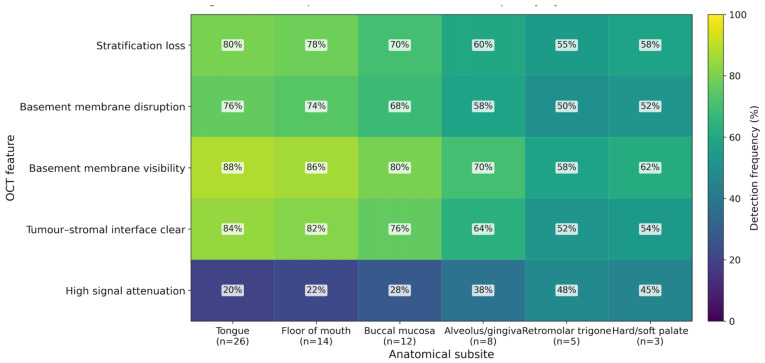
Heatmap of OCT feature detection frequency by anatomical subsite. Heatmap showing the frequency (%) with which key OCT features were identified across oral subsites. Higher detection frequencies for stratification loss, basement membrane disruption/visibility, and clear tumour–stromal interface are seen in anterior regions (tongue and floor of mouth), with reduced detection in posterior and/or keratinised sites (retromolar trigone and palate/gingiva). High signal attenuation demonstrates the opposite pattern, increasing in subsites where optical penetration and interpretability are typically reduced.

Lesion subsite influenced diagnostic interpretability and ROC performance (Figure 5 and Figure 6). Performance was highest for the tongue (AUC 0.97; 95% CI: 0.92–1.00; n = 26) and the floor of the mouth (AUC 0.96; 95% CI: 0.89–1.00; n = 14), followed by the buccal mucosa (AUC 0.93; 95% CI: 0.83–1.00; n = 12) and alveolus/gingiva (AUC 0.90; 95% CI: 0.75–1.00; n = 8). Lower performance was observed in posterior/keratinised regions, including the retromolar trigone (AUC 0.86; 95% CI: 0.65–1.00; n = 5) and hard/soft palate (AUC 0.89; 95% CI: 0.64–1.00; n = 3).

This pattern mirrored subsite-associated differences in OCT feature detection and signal behaviour (Figure 6), with higher detection of stratification loss, basement membrane visibility/disruption, and a clear tumour–stromal interface in anterior subsites and increased signal attenuation in posterior/keratinised subsites, which reduced confidence in feature detection and lesion classification. Given the small numbers in some posterior subsites (notably palate and retromolar trigone), subsite comparisons were interpreted descriptively and were not subjected to formal hypothesis testing.

## 4. Discussion

This prospective clinical study evaluated OCT in the characterisation of 68 histologically confirmed OSCC lesions, incorporating 30 histologically negative adjacent mucosa samples from the same patients to permit estimation of specificity within the same clinical cohort. To our knowledge, this represents one of the larger single-centre OCT series focused specifically on confirmed OSCC, spanning multiple anatomical subsites and differentiation grades. The findings support OCT as a real-time, non-invasive adjunct that can demonstrate microarchitectural features associated with invasion, provide quantitative estimates relevant to tumour assessment, and show grade-associated imaging patterns that may assist lesion stratification.

The diagnostic performance of OCT for invasive OSCCs in this cohort was high. Using the 98-lesion diagnostic dataset (68 OSCC; 30 adjacent mucosa comparators), OCT achieved 98.5% sensitivity, 96.7% specificity, and an AUC of 0.98. The high sensitivity suggests OCT can identify invasive disease in the majority of clinically suspicious lesions, while the specificity achieved against paired adjacent mucosa comparators supports discriminatory capacity in a within-cohort comparison. However, the adjacent mucosa does not capture the full spectrum of benign, inflammatory, and dysplastic mimics encountered in routine practice, and specificity may be lower in broader comparator populations. These results are consistent with earlier studies reporting that invasion-associated OCT patterns—particularly epithelial architectural disruption, reduced basement membrane visibility, and altered subepithelial signal behaviour—are informative for malignancy detection [9,11,12,13,14]. Importantly, the single false negative case and the most common false positive feature in our series highlight known interpretive pitfalls in keratinised or attenuating tissue, where pseudo-loss of basement membrane integrity and reduced visualisation can mimic or obscure invasion. This reinforces the need for structured interpretation criteria and explicit consideration of common artefactual patterns, particularly in sites prone to hyperkeratosis or inflammation-related scattering.

Comparatively, studies evaluating mixed cohorts that include oral potentially malignant disorders and benign lesions typically report lower specificity and wider performance ranges, reflecting a more challenging discrimination task and the risk of spectrum effects. For example, earlier clinical series and systematic reviews have reported variable sensitivity/specificity for OCT in oral carcinogenesis stages, particularly when dysplasia and reactive lesions are included [9,14]. Recent work combining OCT with structured, site-coded acquisition and/or automated image analysis has also shown promise for improving robustness across lesion types and settings [15,16,17]. Taken together, the present findings add to the evidence base by focusing on histologically confirmed invasive OSCC, providing grade-associated imaging biomarker patterns, reporting subsite-specific uncertainty, and operationalising superficial (lateral) margin and depth assessments within known OCT penetration constraints.

Beyond binary classification, OCT provided clinically relevant morphological information linked to tumour differentiation grade. Imaging biomarkers varied systematically across well, moderately, and poorly differentiated OSCCs. Well-differentiated tumours tended to show a thicker epithelium with partial preservation of stratification and relatively frequent basement membrane visibility, whereas poorly differentiated tumours demonstrated near-complete stratification loss, marked basement membrane disruption, high subepithelial reflectivity scores, and high attenuation with reduced visualisation of the tumour–stromal interface. These patterns are biologically plausible: progressive dedifferentiation is expected to disrupt epithelial organisation and tissue interfaces, increase optical heterogeneity, and worsen signal penetration. From a clinical perspective, the ability to detect grade-associated patterns non-invasively may be valuable for triage and biopsy targeting and—if validated in larger studies—could inform pre-MDT planning by signalling lesions more likely to warrant expedited staging, broader mapping biopsies, or wider attention to subsite-related interpretability constraints. Any potential downstream impact on surgical planning would therefore be indirect (via earlier risk stratification and better-targeted sampling), and OCT-derived grade patterns were not used to direct operative decisions in this study; histopathology remains the definitive basis for tumour grading.

OCT also showed utility for quantitative assessment. Bland–Altman analysis demonstrated acceptable agreement between OCT and histology for tumour depth measurement, with a small positive bias indicating slight overestimation by OCT. A tendency towards overestimation is plausible in optical imaging where scattering, surface irregularity, and interpretation of the invasion front can inflate depth estimates, particularly in inflamed or keratinised mucosa. Nonetheless, the observed agreement supports the concept that OCT can contribute quantitative information relevant to lesion assessment, recognising that penetration depth remains limited; therefore, deep margin assessment beyond ~2 mm is not feasible with this approach and deeper invasion cannot be reliably characterised with OCT alone. Visual inspection of the Bland–Altman plot did not suggest a strong proportional bias across the observed depth range, although the number of lesions with depths beyond OCT penetration limits was limited and deep invasion cannot be reliably quantified with OCT.

The anatomical subsite emerged as a practical determinant of OCT interpretability and performance. Subsite-specific analysis showed superior diagnostic performance patterns in the tongue and the floor of the mouth, with reduced performance in posterior and/or keratinised regions such as the retromolar trigone and palate/gingiva. This is consistent with fundamental optical limitations: keratinisation, increased mucosal thickness, dense fibrous stroma, and motion-related instability can reduce signal penetration and degrade the delineation of key interfaces. These findings support the development of subsite-specific acquisition protocols, routine image quality scoring, and interpretation guardrails that explicitly account for keratinisation and attenuation. They also strengthen the case for digital decision support approaches, where automated feature extraction and quality metrics could standardise interpretation across operators and clinical settings.

Several limitations should be considered when interpreting these findings. First, this is a single-centre study using a single OCT platform, and the generalisability of performance across devices, settings, and operator experience requires further evaluation. Second, although adjacent mucosa comparators allow estimation of specificity within the same patient cohort, this comparator is not equivalent to a diverse benign-lesion control population; specificity estimates may differ when OCT is used to discriminate OSCC from a wider range of inflammatory, dysplastic, or reactive lesions encountered in routine practice. Third, OCT interpretation remains operator-dependent. While the technique is rapid and repeatable, consistent acquisition and feature recognition require training, and performance may vary across centres without standardised protocols and competency frameworks. Fourth, OCT penetration depth (typically ~1.5–2 mm) limits assessment of deeper tumour invasion, perineural invasion, and nodal disease, which remain reliant on histopathology and cross-sectional imaging.

In addition, histological dimensions may be affected by tissue processing (including formalin fixation), and a specimen-specific contraction factor was not available in this study; this may contribute to systematic differences between in vivo OCT and histology-derived measurements.

Future work should prioritise multicentre validation with predefined scoring systems, reporting of interobserver reliability, and prospective evaluation against broader clinical comparators, including dysplasia and benign mimics. Integration with digital interpretation platforms, including machine learning models trained on labelled OCT–histology datasets, may reduce subjectivity and improve consistency in challenging subsites. Multimodal approaches—such as combining OCT with fluorescence, photoacoustic imaging, or intraoperative ultrasound—also warrant investigation to mitigate depth limitations and improve margin and invasion assessment in keratinised or posterior regions.

## 5. Conclusions

In this prospective cohort, OCT showed high lesion-level diagnostic accuracy for invasive OSCC and demonstrated grade- and subsite-associated imaging patterns, with clinically useful agreement for superficial (lateral) margin delineation and tumour depth estimation within known penetration limits. OCT should be considered an adjunct to, not a replacement for, biopsy and cross-sectional imaging. Multicentre studies including broader benign and dysplastic comparators and standardised acquisition and interpretation protocols are required before wider clinical implementation. In current practice, OCT’s most immediate utility is as an adjunct to targeted biopsy site selection and superficial margin mapping rather than a standalone diagnostic replacement.

## Figures and Tables

**Table 1:** Patient demographics and clinical characteristics. Summary of demographic details, risk factors, and comorbidities for the 68 patients enrolled in the OCT assessment study.

Characteristic	Value	Characteristic	Value
Number of patients	68	Diabetes mellitus	15 (22.1%)
Age (mean ± SD), years	59.2 ± 10.3	Hypertension	20 (29.4%)
Age range, years	34–83	Cardiovascular disease	12 (17.6%)
Gender (male/female)	40/28	Chronic kidney disease	4 (5.9%)
Smoking status (current/former/never)	30/20/18	Immunosuppression (e.g., steroids, HIV)	3 (4.4%)
Alcohol use (yes/no)	42/26	Family history of head and neck cancer	9 (13.2%)
Betel nut use (yes/no)	10/58	HPV-positive status (where tested)	6/20 tested (30.0%)

**Table 2:** Lesion characteristics and histopathological grades. Detailed description of clinical features, anatomical distribution, lesion morphology, and histopathological differentiation of the 68 OSCC lesions.

Variable	Value	Variable	Value
Total lesions evaluated	68	Clinical appearance of lesions	
Mean lesion size (mm ± SD)	12.4 ± 4.7	Ulcerated	34 (50.0%)
Size range (mm)	5–28	Exophytic	20 (29.4%)
		Indurated	10 (14.7%)
Most common anatomical sites		Mixed morphology	4 (5.9%)
Tongue	26 (38.2%)		
Floor of mouth	14 (20.6%)	Histopathological grade	
Buccal mucosa	12 (17.6%)	Well differentiated	28 (41.2%)
Alveolus/gingiva	8 (11.8%)	Moderately differentiated	30 (44.1%)
Retromolar trigone	5 (7.4%)	Poorly differentiated	10 (14.7%)
Hard/soft palate	3 (4.4%)	Perineural invasion present	19 (27.9%)
		Lymphovascular invasion present	12 (17.6%)

**Table 3:** Diagnostic performance metrics of OCT compared to histopathology. Summary of sensitivity, specificity, predictive values, overall accuracy, area under the ROC curve, and common OCT artefacts in diagnosing invasive OSCCs.

Parameter	Value	Parameter	Value
Total number of lesions (diagnostic dataset)	98	Positive predictive value (PPV)	98.5%
Histologically confirmed invasive OSCC (disease-positive)	68	Negative predictive value (NPV)	96.7%
Histologically negative adjacent mucosa (disease-negative) *	30	Accuracy	98.0%
True positives (TPs)	67	Area under the ROC curve (AUC) **	0.98 (95% CI: 0.96–1.00)
False negatives (FNs)	1	Number of OCT scans with image artefacts	5 (5.1%)
False positives (FPs)	1	Most common false negative subsite	Hard palate/gingiva
True negatives (TNs)	29	Most common false positive feature	Pseudo-loss of BM under hyperkeratosis
Sensitivity	98.5% (95% CI: 92.1–100.0)	Specificity	96.7% (95% CI: 82.8–99.9)

* Disease-negative samples were adjacent mucosa acquired from a subset of the same patients (one sample per patient). ** AUC derived from ROC analysis using an ordinal/continuous OCT score; 95% CI calculated as described in Section 2.5.

**Table 4:** OCT imaging biomarkers stratified by tumour differentiation grade. Comparison of OCT-derived imaging features, including epithelial thickness, basement membrane integrity, stratification patterns, and subepithelial reflectivity across well, moderately, and poorly differentiated OSCCs.

Parameter	Well-Differentiated OSCCs	Moderately Differentiated OSCCs	Poorly Differentiated OSCCs
Number of cases	28	30	10
Mean epithelial thickness (μm)	820 ± 145	610 ± 130	440 ± 110
Thickness range (μm)	610–1100	450–850	310–580
Stratification loss	21.4%	63.3%	100%
BM visibility *	82.1%	42.3%	10.0%
BM disruption *	17.9%	57.7%	90.0%
Subepithelial reflectivity (mean score, 5-point scale)	2.3 ± 0.8	3.6 ± 0.9	4.6 ± 0.5
Subepithelial signal attenuation	Low to moderate	Moderate	High
OCT visualisation of tumour–stromal interface	Clear in 85.7%	Clear in 60.0%	Clear in 30.0%
Image clarity rating (0–5)	4.2	3.5	2.8
Most common artefact type	Surface keratinisation	Inflammation-related scattering	Motion artefact

* BM visibility = presence of a discernible BM-like linear interface on OCT. BM disruption = focal discontinuity/irregularity of this interface where visible. Reduced visibility may reflect optical attenuation (e.g., keratinisation, scattering) and does not indicate absence of histological invasion; all tumours were invasive OSCCs on histopathology.

## Data Availability

The data that support the findings of this study are available upon request from the corresponding authors (W.J. and Z.H.).
